# The Efficacy and Safety of Antifungal Agents for Managing Oral Candidiasis in Oncologic Patients: A Systematic Review With Network Meta-Analysis

**DOI:** 10.7759/cureus.69340

**Published:** 2024-09-13

**Authors:** Amanda F de Lima, Vitor L Fagundes, Nathália B Marques, Helena L Borba, Eric L Domingos, Fernanda S Tonin, Roberto Pontarolo

**Affiliations:** 1 Department of Pharmacy, Federal University of Paraná, Curitiba, BRA; 2 Health and Technology Research Center, Escola Superior de Tecnologia da Saúde de Lisboa (ESTeSL) - Instituto Politécnico de Lisboa (IPL), Lisbon, PRT

**Keywords:** antifungal agents, network meta-analysis, oncologic care, oral candidiasis, treatment choices

## Abstract

This study aimed at synthesizing the available evidence on the comparative safety and efficacy of antifungal agents for preventing or treating oral candidiasis (OC) in oncologic patients. A systematic review following international recommendations was performed (PROSPERO CRD42024507745). A comprehensive search was conducted in PubMed, Scopus, and Web of Science (Feb 2024) to retrieve randomized controlled trials evaluating the clinical effects of antifungal agents in the management of OC in this vulnerable population. Network meta-analyses were performed to evaluate the most prevalent outcomes, with findings reported as odds ratios (ORs) with 95% confidence intervals (CIs). Overall, 24 trials were included, of which 10 addressed OC treatment and 14 disease prophylaxis (n=3449 patients). Fluconazole had the most significant rates of clinical cure when compared to placebo (OR 0.09 [95% CI 0.01-0.69]), amphotericin B (0.21 [95% CI 0.07-0.65]) and itraconazole (OR 0.58 [95% CI 0.34-0.99]); ketoconazole was also superior to placebo for this outcome (OR 0.10 [95% CI 0.03, 0.36]). All antifungal agents presented significantly higher rates of prophylaxis success compared to the absence of an active agent. While these therapies were generally considered safe, only four studies provided data on adverse events, primarily related to gastrointestinal issues. In oncologic patients, azoles (fluconazole, ketoconazole) should be used as a first-line approach for OC treatment. The selection of antifungal agents for disease prophylaxis should consider, among others, patients’ clinical characteristics and preferences. Economic and quality of life-related outcomes should be further addressed in future studies.

## Introduction and background

Oral candidiasis (OC), also known as "thrush" is characterized by excessive fungal growth and superficial invasion of the tissue, affecting the tongue and other areas of the oral mucosa [1]. OC is often a consequence of underlying health conditions such as cancer, HIV, denture use, diabetes, chemotherapy, and corticosteroid therapies, which are associated with states of immunosuppression [2].

In cancer patients, the immune system is suppressed, resulting in diminished defense responses which induces systemic and local alterations, such as a decrease in salivation and damage to oral tissues (e.g., stomatotoxicity). In fact, about 70% of patients undergoing antineoplastic treatment have oral complications that may lead to mucosal colonization by bacteria, fungi, and viruses [3]. Candida spp., a fungus typically found within the oral microbiota of healthy individuals in its benign yeast-like state, can transition into a pathogenic filamentous form (hyphae) under specific conditions and predisposing factors such as immunosuppression [4]. Studies indicate an incidence of candidiasis ranging from around 10% to up to 52% among cancer patients, with strains of Candida spp. resistant to antifungal treatment contributing to high morbidity and mortality rates within this population, including cancer treatment complications [5].

Treatment of OC in cancer patients is challenging due to the limited range of available antifungal agents and scarcity of specific evidence-based guidelines. The main classes include polyenes (e.g., nystatin, amphotericin B) and azoles (e.g., clotrimazole, fluconazole); nevertheless, other treatments lacking specific indication for OC (e.g., terbinafine) can be used particularly in patients experiencing recurrent infections, such as those with HIV [6]. Selecting the most appropriate treatment is further complicated by concerns about drug toxicity, potential interactions, antifungal resistance, and treatment costs. For instance, amphotericin B, while effective, is associated with severe nephrotoxicity, which can be exacerbated when used alongside other nephrotoxic drugs. Similarly, ketoconazole has been linked to serious, sometimes fatal, hepatotoxicity and nephrotoxicity. Specific documented resistance to antifungal drugs, such as fluconazole resistance in Candida species (e.g., mechanisms of mutations in the ERG11 gene and overexpression of efflux pumps), may reduce the efficacy of standard therapies, leading to more persistent and severe infections in this vulnerable population. Moreover, oncologic patients often need second-line antifungal agents or combination therapies, which not only increase direct drug costs but also extend treatment duration, leading to a significant financial burden on both patients and healthcare systems [4-6]. 

Although clinical trials on different antifungal agents for preventing or treating OC are available, comprehensive systematic reviews with meta-analysis that offer an unbiased synthesis of all relevant evidence toward informed decision-making in clinical practice and policy are scarce in the literature. Research in this field is primarily confined to conventional pairwise meta-analyses [7], often limited to assessing few classes of treatments, focusing either on cutaneous or invasive candidemia or restricted to some co-infections. Moreover, their results are frequently inconclusive to claim or refute the benefit of any antifungal agent in treating candidiasis in these patients [8-12]. Few network meta-analyses, a technique that has the advantage of simultaneously combining both direct evidence (i.e., available in the literature) and indirect evidence (i.e., estimated based on common treatment comparators) across several treatments in a single model, are available in this field [7]. Moreover, existing studies are often outdated (the last searches were performed in 2018) and are limited to one approach to manage OC [13,14]. Given that the efficacy, safety profile, and cost-effectiveness of antifungal therapies may differ significantly, particularly in immunocompromised populations like cancer patients, there is a critical need for broader systematic comparisons of these agents to determine the most effective and safe options for this vulnerable group and ground the development of updated clinical practice guidelines. 

Therefore, the aim of this study was to synthesize the existing evidence on the comparative clinical profile of all antifungal agents used both in the treatment and prophylaxis of OC in cancer patients.

## Review

Material and methods

Search Strategy and Eligibility Criteria

This study was performed in accordance with the Cochrane Collaboration recommendations and reported following the Preferred Reporting Items for Systematic Reviews and Network Meta-Analyses (PRISMA-NMA) [15]. The study is registered in PROSPERO under the number CRD42024507745. Two authors independently conducted all steps of the studies’ selection (screening and eligibility), data extraction, and risk of bias assessment. A third reviewer was consulted in case of discrepancies.

The bibliographic search for clinical trial references was carried out on February 15, 2024 in PubMed, Scopus, and Web of Science, without date (from database inception) or language restrictions (no filters were applied). Descriptors such as 'candidiasis', 'antifungal agents', 'clinical trials', and 'cancer' were used; the full search strategy for each database is available in the Appendix. Reference lists of included studies and clinical trial registration databases (clinicaltrials.gov) were also manually searched (see complete search strategy in the Appendix). Two researchers independently screened titles and abstracts of the retrieved registers. Relevant records were then evaluated in full; primary studies that met the following inclusion criteria (PICOS acronym) were included for data extraction and synthesis: population: studies evaluating oncologic patients (diagnosed with any type of cancer) of any age and sex with indication for prophylaxis or treatment for OC; interventions: studies assessing the use of antifungal agents administered topically or systemically, regardless of their pharmaceutical form and dosage to manage or prevent OC; comparators: studies with any antifungal intervention or placebo as control; outcomes: studies assessing at least one of the following outcomes related to prophylaxis (prophylaxis success; rate of OC infections), disease treatment (clinical cure as resolution or improvement of symptoms; overall cure reported as complete response or significant improvement of lesions); overall safety (as most reported adverse events (e.g., nausea, vomiting), treatment discontinuation, death); study design: randomized controlled trials (RCTs) (i.e., primary studies with the highest level of evidence to inform clinical practice). Studies written in non-Roman characters, those focused on other objectives such as pharmacokinetics (i.e., pharmacological behavior of a drug instead of clinical outcomes of treatments in a controlled setting), or those restricted to economic or humanistic outcomes (without clinical data) were excluded. No studies were excluded based on the sample size or methodological quality. 

Data Extraction and Risk of Bias Assessment

A standardized spreadsheet was used to extract information from the included studies: overall data (authors, year of publication, conflict of interest, country, study duration, sample size); participants’ characteristics (age, sex, diagnosis); details of the intervention and controls (drugs, dose, route of administration, regimen); clinical outcomes results.

The risk of bias in the included studies was evaluated using the Cochrane Collaboration tool for assessing the risk of bias in randomized trials of interventions (RoB 2.0) [16]. The tool incorporates the evaluation of the following domains per outcome of interest: (1) Bias arising from the randomization process; (2) Bias due to deviations from intended interventions; (3) Bias due to missing outcome data; (4) Bias in measurement of the outcome; (5) Bias in selection of the reported result. At the end of the assessment, the studies were classified as having “high risk”, “some concerns” or “low risk” of bias. A detailed explanation of the RoB 2.0 use is available in the Appendix. 

Statistical Analyses

A narrative synthesis of the findings from the included studies, structured around the type of intervention, population subgroup (type of cancer), and type of outcomes (prophylaxis and treatment) was provided in tables and the text using descriptive statistics. Additionally, network meta-analysis, an approach recommended by the International Society for Pharmacoeconomics and Outcome Research (ISPOR) given its advantage of providing multiple treatment comparisons across studies was performed for each outcome of interest whenever possible [7,17]. Transitivity analyses were performed by comparing population, interventions, control, and outcome definitions among studies (i.e., qualitative evaluation to confirm data homogeneity). A standard heterogeneity parameter and common effect model (i.e., one true treatment effect for each comparison) were assumed for all comparisons. A consistency model (league table assuming that the direct evidence and indirect evidence in the network are consistent) was built for each outcome, and the treatments’ relative effect sizes were reported as odds ratio (OR) with 95% confidence intervals (CIs). To increase the estimated precision of the relative effect sizes of comparisons and to properly account for correlations between multi-arm trials, rank probabilities involving all treatment options were built for each outcome (data reported as p-score, %). To estimate the robustness of the networks (i.e., consistency check), node-splitting local tests for inconsistency (i.e., the difference between the pooled direct and indirect evidence for a particular comparison) were performed (p-values<0.05 reveal inconsistencies in the network). Network analyses were performed in CINeMA (Confidence in Network Meta-Analysis) and drug ranking in NMAstudio (web applications based on frequentist programming in Python linked to R-package netmeta) [18-20].

Results

**Figure 1:**
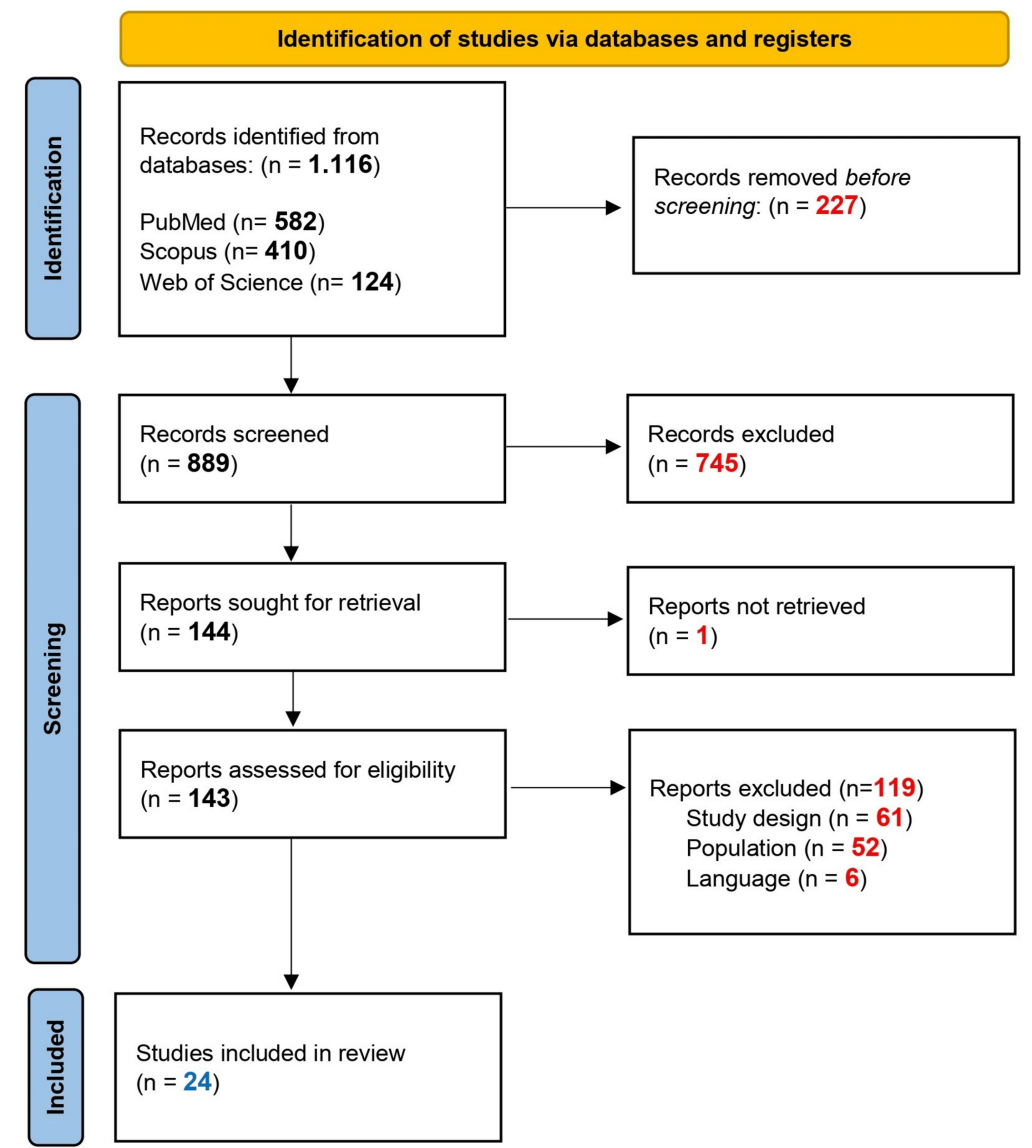
PRISMA flowchart of the systematic review process Source: [45] PRISMA: Preferred Reporting Items for Systematic Reviews and Meta-Analyses

A total of 889 records were retrieved through the systematic search after duplicate removal of which 745 were excluded during screening (i.e., irrelevant to the study). From the 144 assessed by full-text reading, 120 were excluded (52 did not correspond to the population, 61 due to study design and 7 due to language) (see the complete list of excluded studies with reasons for exclusion in the Appendix). Finally, 24 RCTs met the study’s inclusion criteria; 14 (58.3%) referring to OC prophylaxis and 10 (41.6%) to disease treatment (see Figure 1) [21-44]. No additional study was found through manual searches.

These 24 trials (n=3449 patients) were published between 1974 and 2008. Studies on prophylaxis were mostly performed in Europe (n=5 studies; 35.7%), and the USA (n=4 studies; 28.6%); three studies did not report the country of origin, one was carried out in Saudi Arabia and one was a multinational trial (Australia, Belgium, Canada, Germany, Korea, and Spain). Conversely, trials on OC treatment were mostly conducted in the USA (n=5, 50%), followed by European countries such as Belgium (n=2; 13.3%), France (n=1; 6.7%), and Scotland (n=1; 6.7%); one study did not report the country. Fluconazole was the most assessed antifungal - either in prophylactic or therapeutic studies (n=8; 33.3% and n=4; 26.7%, respectively). Nystatin was also commonly used as disease prophylaxis (n=7; 29.2%), while amphotericin B and ketoconazole were fairly assessed as treatments (n=3; 18.8% each). Placebo was the most common comparator, reported in about a third of all studies. The mean duration of prophylaxis and OC treatments was 27 and 17 days, respectively. Most patients were diagnosed with hematological cancer in both prophylaxis and treatment studies (60.0% and 85.7% respectively) followed by head and neck tumors (30.0% and 21.4%) (see Table 1).

**Table 1:** Main characteristics of the included studies for disease prophylaxis (n=14) and treatment (n=10) *Cancer of the head and neck includes malignancies affecting the oral cavity, pharynx, larynx, salivary glands, sinonasal area, skin, and thyroid, as well as metastatic cancers in these regions. Hematologic cancers encompass malignancies of the blood, bone marrow, and lymphatic system, including leukemia, lymphoma, and myeloma. Solid tumors refer to malignancies that form distinct masses of abnormal tissue in organs or tissues, excluding blood cancers (e.g., breast, lung, prostate, and colorectal cancers). AMB: Amphotericin B; AUS: Absence of treatment; CETO: Ketoconazole; CLO: Clotrimazole; ENXAG: Mouthwash; FLU: Fluconazole; ITRA: Itraconazole; MICO Adhesiv: Miconazole adhesive; MICO gel: Miconazole oral gel; NATA: Natamicin; NIS: Nystatin; NIS (SUSP): Nystatin suspension; NR: Not reported; PLA: Placebo; UK: United Kingdom; USA: United States of America.

Author, year	Region	Drug	Dose	Frequency (per day)	Number of randomized patients	Mean age (years)	Female (%)	Treatment duration (days)	Type of cancer*
OC treatment									
Bensadoun et al. 2007 [21]	France	MICO Adhes.	50mg	1	154	53	27	14	Head and neck
		MICO gel	125mg	4	152	54	23	14	
Chen et al. 1974 [22]	USA	AMB	100mg	4	101	60.5	17.82	28	Head and neck
		PLA	-	-				28	
Hughes et al. 1984 [24]	USA	CETO	200mg	1	36	14	NR	14	Hematologic/Solid
		PLA	-	-	20	7	NR	14	
Lake et al. 1997 [25]	USA	FLU	100mg	2	16	53	50	19	NR
		AMB	0.3mg/kg	1	15		33.33	12	
Lawson et al. 1980 [29]	USA	CLO	50mg	5	84	43	48.4	30	Hematologic/Solid
		NIS	50mg	5				30	
Meunier et al. 1990 [26]	Belgium	CETO	200mg	3	18	NR	44.4	23	Hematologic/Solid
		NIS	1000.000U	3	24	NR	41.6	23	
Meunier et al. 1990 [30]	Belgium	FLU	100mg	1	19	NR	46.15	14	Hematologic/Solid
		CETO	40mg	1	18	NR	50	14	
Oude et al. 2004 [27]	Europe	FLU	100mg	1	126	54	53	10	Hematologic/Solid/Lymphoma
		ITRA	200mg	1			44	15	
Finlay et al. 1996 [23]	UK	FLU	50mg	1	37	64	NR	7	Head and neck
		AMB	10mg	4	36		NR	14	
Yap et al. 1980 [28]	USA	CLO	10mg	5	26	44	50	14	Hematologic/Solid/Lymphoma
		CLO	50mg	5				14	
OC prophylaxis									
Corvo et al. 2008 [31]	Italy	FLU	100mg	1	138	61.9	31	37.1	Head and neck
		PLA	-	-	132	61.2	24	33.4	
Egger et al. 1995 [32]	Switzerland	FLU	400mg	1	43	41	53.5	NR	Hematologic
		NIS Susp	24x10^6	3	46	36	45.65	NR	
Ellis et al. 1994 [33]	Saudi Arabia	FLU	200mg	2	42	26	47.62	24	Hematologic
		CLO	10mg	1		21	45.83	19	
		ENXAG	500.000U	4	48	NR	NR	NR	
Groll et al. 1997 [34]	Germany	FLU	3mg/kg	1	25	8.25	48	30	Head and neck/Hematologic/Solid
		NIS	50.000U	4			52	31	
Hansen et al. 1987 [35]	NR	CETO	400mg	1	27	NR	NR	NR	Hematologic
		PLA	-	-	29	NR	NR	NR	
Ninane et al. 1994 [36]	8 countries	FLU	3mg/kg	1	245	6.8	37.9	27.8	Hematologic/Solid
		NIS	50.000U/kg	4	257		43.2	29.2	
		AMB	25mg/kg	4				29.2	
Owens et al. 1984 [37]	USA	CLO	10mg	3	42	46	40.4	NR	Hematologic/Solid
		PLA	-	-		43.5	42.8	NR	
Philpott-Howar et al. 1993 [38]	UK	FLU	50mg	1	269	46.1	41.6	28	Hematologic
		NIS	4x10^6U	1	267	45.7	46	28	
		AMB	2g	1				28	
Scrimgeour et al. 1985 [44]	NR	CETO	200mg	1	12	NR	69.8	21	NR
		CETO	400mg	1	18	NR		21	
Samonis et al. 1990 [39]	United States	FLU	50mg	1	73	57	62	10	Head and neck/Hematologic
		PLA	-	-			51.8	9	
Vogler et al. 1987 [40]	USA	CETO	200mg	2	22	NR	45.4	24	Hematologic
		NIS	100.000U/mL	10mL/4xd	24	NR	37.5	23	
Williams et al. 1977 [41]	UK	NIS	100.00/10mL	every 2h	13	NR	NR	NR	Hematologic
		NATA	0.25%/10mL	every 2h	15	NR	NR	NR	
		AUS	-	-	28	NR	NR	NR	
Yeo et al. 1985 [42]	USA	CLO	10mg	3	153	55	56.56	12	Hematologic/Solid
		AUS	-	-	143		66.66	14	
Young et al. 1999 [43]	6 countries	FLU	100mg	2cps 1x/d	86 78	47.9	52	42	Hematologic
		FLU-PLA	-						
		NIS	100.000U/mL	10mL 6x/d		48.5	36	42	
		NIS-PLA	-						

**Figure 2:**
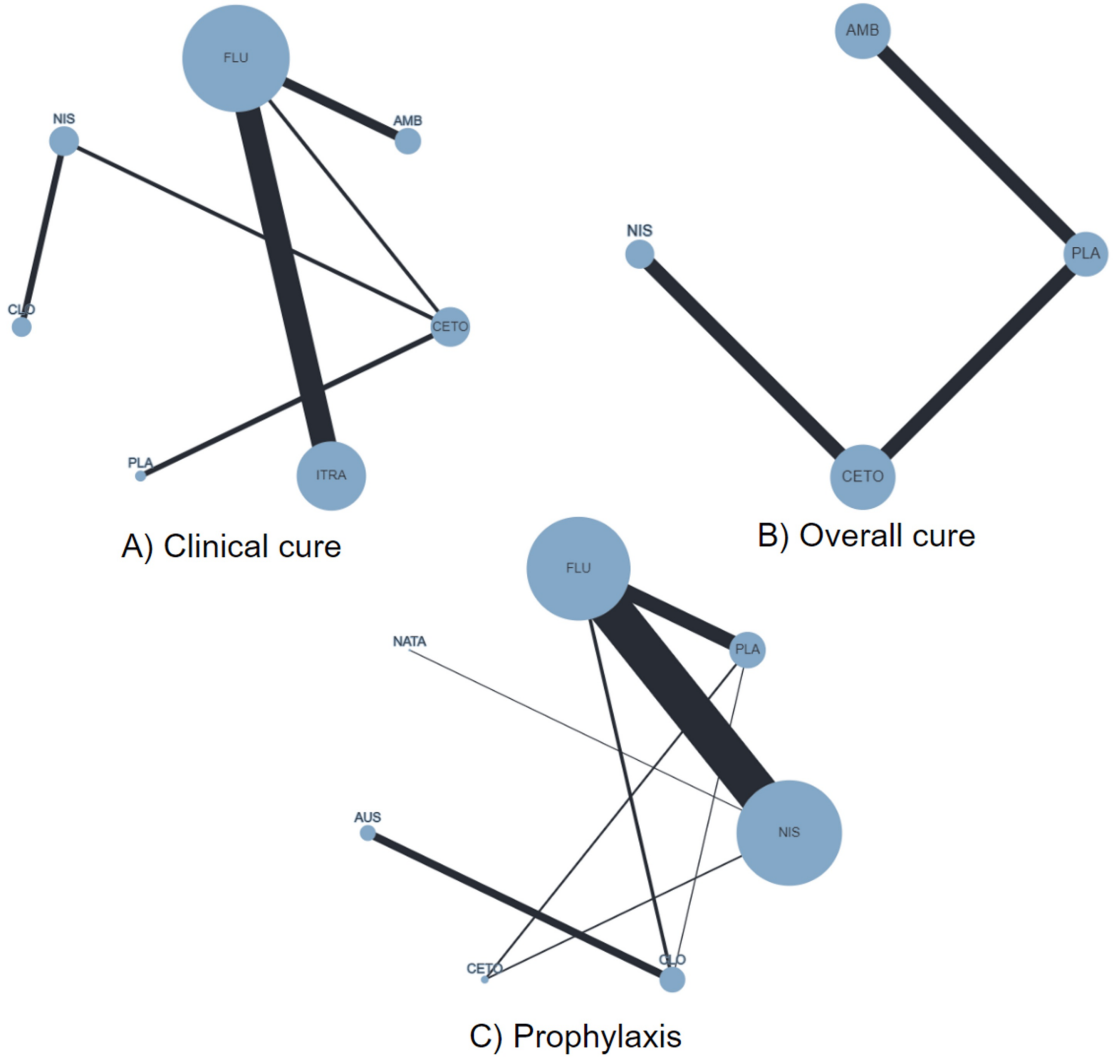
Network meta-analyses plots for the outcomes of interest Note: in the network meta-analysis plot, the size of each circle represents the number of studies evaluating a particular intervention. The lines connecting circles indicate the number of direct comparisons available in the literature, with the thickness of the lines reflecting the number of studies supporting each comparison. AMB: Amphotericin B; AUS: Absence of treatment; CETO: Ketoconazole; CLO: Clotrimazole; FLU: Fluconazole; ITRA: Itraconazole; NATA: Natamicin; NIS: Nystatin; PLA: Placebo.

We were able to construct three network meta-analyses for the primary outcomes reported in the studies: prophylaxis (n=13 studies that evaluated fluconazole, clotrimazole, ketoconazole, nystatin, natamycin, placebo/no treatment), clinical cure (n=7 studies that evaluated fluconazole, clotrimazole, ketoconazole, itraconazole, nystatin, amphotericin B and placebo) and overall cure measured as complete treatment response (n=3 studies that evaluated ketoconazole, nystatin, amphotericin B and placebo) (Figure 2).

In the network of prophylaxis, all antifungal agents (ketoconazole, clotrimazole, fluconazole, natamycin, and nystatin) were statistically more associated with OC prevention when compared to placebo or no treatment (OR varying from 0.01 [95% CI 0.00, 0.88] to 0.03 [95% CI 0.00, 0.34]). However, no other differences were observed between active treatments for this outcome (see Table 2). Yet, according to the treatment ranking, the highest probabilities of disease prevention were obtained for fluconazole (p-score 85%), followed by natamycin and ketoconazole (around 70% each) (see complete ranking in the Appendix). Regarding disease treatment, fluconazole also provided significantly higher clinical cure rates (p-score around 70%) when compared to itraconazole (OR 0.58 [95% CI 0.34-0.99]) (p-score 41%), amphotericin B (0.21 [95% CI 0.07-0.65]) (p-score around 20%) and placebo (OR 0.09 [95% CI 0.01-0.69]) (p-score 5%). Nystatin, clotrimazole, and ketoconazole were also associated with higher rates of clinical cure compared to placebo (p-scores of 86%, 76%, and 57%, respectively) (see Table 3) (complete ranking available in the Appendix). Conversely, the network of overall cure found only amphotericin B to be superior to placebo (OR 0.05 [95% CI 0.00-0.94]), with a probability of leading to this event of around 80%. Nystatin and ketoconazole were ranked second and third options for this outcome (p-scores of 64% and 53%, respectively) (see Table 4; complete ranking available in the Appendix). All original networks were found to be robust within the transitivity analysis (no node-split analyses were possible given the reduced number of studies per comparison in a node and the simple geometry of the networks, e.g., star-shaped).

**Table 2:** Consistency analyses of the network of OC prophylaxis Note: Comparisons between treatments should be read from left to right, and the estimated value is in the cell in common between the column definition treatment and the line definition treatment. Results are presented as odds ratios (OR) with 95% confidence intervals (CIs). An OR <1 favors the column-defining treatment (intervention from the left); an OR>1 favors intervention from the right. AUS: Absence of treatment; CETO: Ketoconazole; CLO: Clotrimazole; FLU: Fluconazole; NATA: Natamicin; NIS: Nystatin; PLA: Placebo.

Drug comparisons						
AUS	--	--	--	--	--	--
0.02 (0.001, 0.59)	CETO	--	--	--	--	--
0.03 (0.002, 0.34)	1.56 (0.13, 18.53)	CLO	--	--	--	--
0.01 (0.001, 0.20)	0.61 (0.08, 4.82)	0.39 (0.09, 1.79)	FLU	--	--	--
0.01 (0.000, 0.88)	0.74 (0.02, 25.46)	0.47 (0.02, 14.15)	1.20 (0.06, 25.48)	NATA	--	--
0.02 (0.001, 0.50)	1.36 (0.19, 10.02)	0.87 (0.15, 4.93)	2.22 (0.93, 5.31)	1.85 (0.10, 34.45)	NIS	--
0.08 (0.004, 1.50)	4.45 (0.52, 37.77)	2.84 (0.59, 13.79)	7.25 (2.26, 23.31)	6.03 (0.23, 155.52)	3.27 (0.79, 13.43)	PLA

**Table 3:** Consistency analyses of the network of OC treatment for clinical cure Note: Comparisons between treatments should be read from left to right, and the estimated value is in the cell in common between the column definition treatment and the line definition treatment. Results are presented as odds ratios (OR) with 95% confidence intervals (CIs). An OR <1 favors the column-defining treatment (intervention from the left); an OR>1 favors intervention from the right. AMB: Amphotericin B; CETO: Ketoconazole; CLO: Clotrimazole; FLU: Fluconazole; ITRA: Itraconazole; NIS: Nystatin; PLA: Placebo.

Drug comparisons						
AMB	--	--	--	--	--	--
4.42 (0.65, 30.22)	CETO	--	--	--	--	--
9.85 (0.60, 161.6)	2.23 (0.29, 17.03)	CLO	--	--	--	--
4.73 (1.75, 14.46)	1.07 (0.22, 5.12)	0.48 (0.04, 6.25)	FLU	--	--	--
2.73 (0.79, 9.42)	0.62 (0.12, 3.23)	0.28 (0.02, 3.81)	0.58 (0.34, 0.98)	ITRA	--	--
11.89 (0.98, 144.1)	2.69 (0.55, 13.20)	1.21 (0.34, 4.28)	2.51 (0.27, 23.40)	4.36 (0.44, 43.26)	NIS	--
0.43 (0.04, 4.37)	0.10 (0.03, 0.36)	0.04 (0.00, 0.49)	0.09 (0.01, 0.69)	0.16 (0.02, 1.29)	0.04 (0.00,0.28)	PLA

**Table 4:** Consistency analyses of the network of OC treatment for overall cure Note: Comparisons between treatments should be read from left to right, and the estimated value is in the cell in common between the column definition treatment and the line definition treatment. Results are presented as odds ratios (OR) with 95% confidence intervals (CIs). An OR <1 favors the column-defining treatment (intervention from the left); an OR>1 favors intervention from the right. AMB: Amphotericin B; CETO: Ketoconazole; FLU: Fluconazole; NIS: Nystatin; PLA: Placebo.

Drug comparisons			
AMB	--	--	--
0.33 (0.00, 12.10)	CETO	--	--
0.42 (0.00, 19.16)	1.27 (0.36, 4.54)	NIS	--
0.05 (0.00, 0.94)	0.16 (0.02, 1.35)	0.12 (0.01, 1.51)	PLA

The therapies were generally considered safe, but only four studies reported adverse event rates, three for antifungal prophylaxis [41,42,44] and one for treatment of OC [22]. The most frequently reported events were nausea, vomiting, overall gastrointestinal discomfort, and treatment discontinuation. The results for these outcomes are briefly summarized in Table 5. No meta-analyses were performed given the scarcity of data and lack of standardized outcome reporting.

**Table 5:** Main adverse effects of the included studies for prophylaxis (n=14) and treatment (n=10) of the disease AMB: Amphotericin B; AUS: Absence of treatment; CETO: Ketoconazole; CLO: Clotrimazole; ENXAG: Mouthwash; FLU: Fluconazole; ITRA: Itraconazole; MICO Adhesiv: Miconazole adhesive; MICO gel: Miconazole oral gel; NATA: Natamicin; NIS: Nystatin; NIS (SUSP): Nystatin suspension; NR: Not reported; PLA: Placebo

Author, year	Drug	Dose	Number of randomized patients	Nausea (n/total)	Vomiting (n/total)	Gastrointestinal events (n/total)	Discontinu-ation (n/total)	Death (n/total)
OC treatment								
Bensadoun et al. 2007 [21]	MICO Adhes.	50mg	154	1/147	1/147	5/147	3/147	3/147
	MICO gel	125mg	152	4/147	3/147	4/147	6/147	3/147
Chen et al. 1974 [22]	AMB	100mg	101	NR	NR	NR	NR	NR
	PLA	-		NR	NR	NR	NR	NR
Hughes et al. 1984 [24]	CETO	200mg	36	3/36	NR	NR	NR	NR
	PLA	-	20	NR	NR	NR	NR	NR
Lake et al. 1997 [25]	FLU	100mg	16	1/16	2/16	NR	NR	NR
	AMB	0.3mg/kg	15	1/15	2/15	NR	NR	NR
Lawson et al. 1980 [29]	CLO	50mg	84	3/36	NR	NR	NR	11/66
	NIS	50mg		20/30	NR	NR	NR	
Meunier et al. 1990 [26]	CETO	200mg	18	NR	NR	NR	NR	2/18
	NIS	1000.000U	24	NR	NR	NR	NR	3/24
Meunier et al. 1990 [30]	FLU	100mg	19	1/18	NR	NR	2/18	NR
	CETO	40mg	18	NR	NR	NR	NR	NR
Oude et al. 2004 [27]	FLU	100mg	126	NR	NR	NR	NR	17/126
	ITRA	200mg	126	NR	NR	NR	NR	22/126
Finlay et al. 1996 [23]	FLU	50mg	37	NR	NR	NR	NR	NR
	AMB	10mg	36	NR	NR	NR	NR	NR
Yap et al. 1980 [28]	CLO	10mg	26	1/26	NR	1/26	NR	NR
	CLO	50mg			NR		NR	NR
OC prophylaxis								
Corvo et al. 2008 [31]	FLU	100mg	138	13/138	NR	7/138	NR	NR
	PLA	-	132	4/132	NR	7/132	NR	NR
Egger et al. 1995 [32]	FLU	400mg	43	NR	NR	NR	1/43	NR
	NIS Susp	24x10^6	46	NR	3/46	NR	3/46	NR
Ellis et al. 1994 [33]	FLU	200mg	42	NR	NR	NR	NR	2/42
	CLO	10mg		NR	NR	NR	NR	9/48
	ENXAG	500.000U	48	NR	NR	NR	NR	NR
Groll et al. 1997 [34]	FLU	3mg/kg	25	1/25	NR	4/25	NR	NR
	NIS	50.000U	25	NR	NR	NR	NR	NR
Hansen et al. 1987 [35]	CETO	400mg	27	NR	NR	NR	2/27	NR
	PLA	-	29	NR	NR	NR	NR	NR
Ninane et al. 1994 [36]	FLU	3mg/kg	245	NR	NR	27/245	NR	NR
	NIS	50.000U/kg	257	NR	NR	16/257	NR	NR
	AMB	25mg/kg	257	NR	NR	16/257	NR	NR
Owens et al. 1984 [37]	CLO	10mg	42	1/42	NR	NR	NR	NR
	PLA	-		NR	NR	NR	NR	NR
Philpott-Howar et al. 1993 [38]	FLU	50mg	269	2/269	1/269	7/269	7/269	NR
	NIS	4x10^6U	267	6/267	7/267	12/267	7/267	NR
	AMB	2g						
Scrimgeour et al. 1985 [44]	CETO	200mg	12	NR	NR	NR	NR	NR
	CETO	400mg	18	NR	NR	NR	NR	NR
Samonis et al. 1990 [39]	FLU	50mg	73	1/58	1/58	NR	3/58	NR
	PLA	-		NR	NR	NR	NR	NR
Vogler et al. 1987 [40]	CETO	200mg	22	NR	NR	NR	NR	NR
	NIS	100.000U/mL	24	NR	1/24	NR	NR	NR
Williams et al. 1977 [41]	NIS	100.00/10mL	13	NR	NR	NR	NR	NR
	NATA	0.25%/10mL	15	NR	NR	NR	NR	NR
	AUS	-	28	NR	NR	NR	NR	NR
Yeo et al. 1985 [42]	CLO	10mg	153	0/153	0/153	0/153	0/153	0/153
	AUS	-	143	0/143	0/143	0/143	0/143	0/143
Young et al. 1999 [43]	FLU	100mg	86	5/85	4/85	16/85	NR	6/85
	FLU-PLA	-						
	NIS	100.000U/mL	78	6/78	9/78	17/78	NR	11/78
	NIS-PLA	-						

The overall risk of bias for the main outcomes of interest (prophylaxis, clinical cure, overall cure, and overall safety) was assessed as moderate. Among the studies evaluating prophylaxis, over half (57%) were found to have a high risk of bias, while about 43% exhibited some methodological concerns. For studies on OC treatment, the risk of bias varied by outcome: 40-50% of studies had some important concerns, while 20-40% presented a high risk of bias. Key methodological weaknesses were identified for the domains of outcomes measurement (especially regarding subjective data reported by the patient) and randomization (i.e., allocation process was often poorly reported). See the Appendix for detailed evaluation.

Discussion

This systematic review with network meta-analysis synthesized the evidence from 24 clinical trials on the comparative efficacy and safety of seven different antifungal agents addressing both prophylactic and treatment strategies for managing OC in oncologic patients. Our analysis found that azoles, particularly fluconazole, clotrimazole, and ketoconazole are the most promising therapies for treating this population. Additionally, fluconazole and natamycin demonstrated superior prophylactic effects in preventing disease infection. Candidiasis of oral mucosa (caused mostly by C. albicans), oral herpes simplex, and oral mucositis are the most common oral cavity infections in immunosuppressed patients. Their prevalence may reach 50% of all cancer cases before treatment, with higher rates in head and neck cancer group (especially oral and maxillofacial tumors (around 60-70%)) followed by hematological malignancy group (50-60%), which was also observed in our review [46-48]. Research suggests that in addition to the immunosuppression, salivary gland dysfunction, and poor oral hygiene among these patients, besides irradiation-induced histological alterations contribute to the development of oral mucositis and create an environment for yeast proliferation [49-51]. Also, it has been proposed that Candida species can produce carcinogenic compounds (e.g., nitrosamines), increase the level of certain enzymes (e.g., metalloproteinases), and promote angiogenesis, favoring progression and metastatic potential [48,52].

Given that clinical OC typically presents with pseudomembranous lesions (resembling white oral thrush), mucosal redness, chronic oral pain, and a burning sensation within the oral cavity, prompt access to therapeutic strategies is paramount [53]. Despite the reduced number of included studies reporting reliable data (i.e., few studies with moderate-high quality) in our treatment network meta-analysis, findings suggest some azoles as more effective in eradicating OC. Conversely, echinocandins such as amphotericin B presented poorer results and thus should be avoided as a first-line approach. Previous scattered meta-analyses, yet focusing on immunosuppressed patients (e.g., HIV-infected adults, oncologic cases), similarly demonstrated fluconazole as having a superior clinical profile compared to other agents (p<0.01; ranking first at the surface under the cumulative curve (SUCRA) analysis ranging between 79-87% probability) [8,13,54]. In fact, a susceptibility study conducted by Monsen et al. (2023) showed that 98% of C. albicans isolates were susceptible to fluconazole [55]. This fungistatic azole inhibits the biosynthesis of ergosterol by interfering with the fungal enzyme lanosterol-demethylase and has a straightforward intestinal absorption and oral bioavailability equivalent to 90% of that attained through intravenous administration [56,57]. Other studies additionally demonstrated that ketoconazole is more effective than placebo in treating OC (RR 3.61 [95% CI 1.47-8.88]), especially when evaluated through mycological assessment [13,58].

Although some discussions on more readily available evidence on preventing OC may exist in the literature, consensus on the best approach remains elusive. This scarcity of evidence on the topic in vulnerable populations across different geographical regions underscores the urgent need for further research to address these gaps and improve patients' clinical outcomes. Yet, similarly to our findings, the meta-analyses by Shen Loo et al. (2021) demonstrated most antifungal agents (amphotericin B, clotrimazole, fluconazole, itraconazole, ketoconazole, miconazole, natamycin, nystatin, and combinations of nystatin/miconazole and nystatin/amphotericin B) as having some beneficial prophylactic effect against OC when compared with placebo [14]. Authors additionally suggested the potential of clotrimazole as the best agent for reducing the risk of OC compared to placebo (RR 0.21 [95% CI 0.08 to 0.55]; SUCRA 88.5%), followed by fluconazole (RR 0.34 [95% CI 0.18 to 0.66]; SUCRA 78.2%), amphotericin B (RR 0.51 [95% CI 0.27 to 0.96]; SUCRA 61.8%) and ketoconazole (RR 0.55 [95% CI 0.34 to 0.92]; SUCRA 60%). Yet, no differences among active therapies were found [14]. On the other hand, the meta-analyses by Ramírez-Carmona et al. (2023), including both randomized and non-randomized trials, recommend fluconazole as the first-line approach for the prophylaxis of any oral fungal diseases (RR 0.30 [95% CI 0.16-0.55] vs placebo in randomized trials), being potentially more effective than amphotericin B and nystatin (p<0.01 and p=0.02, respectively) [4]. The certainty of the evidence was rated as low to very low due to the significant risk of bias and methodological concerns in the studies. However, this does not necessarily compromise the reliability of the findings. It underscores the need for well-designed trials in this field, directly comparing key drugs of interest (e.g., fluconazole vs. other azoles). These limitations also call for cautious interpretation of the results within the context of the existing evidence. 

All the evaluated agents seem to be safe for treating or preventing OC, which corroborates with the findings from Ramírez-Carmona et al. (2023) reporting no significant differences between antifungal agents, including different azoles, echinocandins, and their combinations [4]. Fluconazole was ranked as the safest among other antifungal agents (SUCRA 80%), whereas clotrimazole (SUCRA 36%) and amphotericin B (SUCRA 18%) ranked low for safety by Shen Loo et al. (2021) [14]. As in other areas, amphotericin B is associated with the highest risk of adverse events, including nephrotoxicity [7,10,59]. Yet, due to the scarce safety data reported in the primary studies, no meta-analyses were possible, preventing further conclusions for the management of OC in cancer patients. No specific reasons for this under-reporting of adverse drug reactions were found, but potential factors may include the study’s year of publication (e.g., before the establishment of international guidelines for conducting and reporting RCTs), the short duration of treatment periods, limited follow-up time, or an emphasis on primary efficacy outcomes over safety data. Additionally, smaller sample sizes or insufficient monitoring for adverse effects may have contributed to incomplete reporting. 

While some international guidelines on the management of fungal infection are available, they may not focus exclusively on OC in oncologic patients, which may hamper tailored decision-making. According to the Infectious Diseases Society of America (ISDA) recommendations, the current first choice for the treatment of OC in patients free from other conditions (e.g., neutropenia or cancer), are azoles (clotrimazole, miconazole) or even nystatin [56]. In our network meta-analysis, these drugs did not demonstrate significantly superior efficacy compared to others for either preventing or managing OC in patients with malignancies. This lack of distinction may be attributed, in part, to the unique vulnerabilities and complexities of this population. In fact, when it comes to clinical practice recommendations for moderate to severe diseases or for patients with recurrent infections, oral fluconazole is usually the first choice in a 7 to 14 days treatment regimen [56]. Other medications, such as amphotericin B and itraconazole can be used for refractory infections or as a second option in cases of contraindication to fluconazole [56,60]. The findings underscore the need for further research to provide more tailored data, including treatment algorithms, dosing patterns, and regimens, as well as clinical recommendations that address the specific needs of oncologic patients. Cost-effectiveness analyses should also be performed to evaluate the economic impact of different treatment strategies and ensure that recommendations are not only clinically effective but also economically viable.

Our study has some limitations. The poor reporting quality of some studies prevented further statistical analyses on the comparative effects of antifungal agents. The systematic review was carried out following international recommendations, with searches performed in three databases to maximize retrieving studies; nonetheless, given the scattering of publications across the biomedical field (from dentistry to oncology), it is possible that some literature was not recovered. Yet, manual searches were performed to fill this gap. Due to limitations in assessing articles written in non-Roman characters, some literature may be missing, potentially introducing a geographical bias. However, it is important to note that the majority of scientific articles are published in English. As in some studies, patients underwent oncological treatment (e.g., chemotherapy, radiotherapy), it is difficult to confirm whether the observed adverse effects were related to the antifungal use. Thus, caution is recommended when interpreting these findings and translating them into clinical practice. Like any advanced method, network meta-analysis has its limitations. To simplify data interpretation, we employed a frequentist network meta-analysis using a single, uniform estimate of heterogeneity across all treatment comparisons. This approach is commonly recommended for small networks, though it may not capture variation in heterogeneity across different comparisons [17]. The validity of the analysis also depends on how treatment effects vary across different comparisons; therefore, treatment rankings should be interpreted in the context of these relative effects. 

## Conclusions

For the treatment of OC in oncologic patients, azoles remain the recommended first-line therapy, with clotrimazole, fluconazole, and ketoconazole being the preferred agents according to our findings. Additionally, clotrimazole and fluconazole presented promising efficacy in preventing OC, though no statistical differences were observed among antifungal agents. However, due to variations in dosages and treatment durations across the included studies, we were unable to determine the optimal dosing regimens for different cancer types or stages. This highlights the need for tailored recommendations based on patient characteristics and cancer progression, as well as further studies to establish standardized treatment protocols. While we also emphasize the need for further research on economic and quality-of-life outcomes in this field, these factors already play a significant role in shaping treatment decisions and patient care. Patients' clinical needs as well as drugs' preferences, accessibility, and affordability are critical in oncology, where prolonged therapy and multiple interventions are common. Future research should aim to address these aspects to ensure more target evidence-based recommendations for managing OC in cancer patients. 

## Figures and Tables

**Figure 3:**
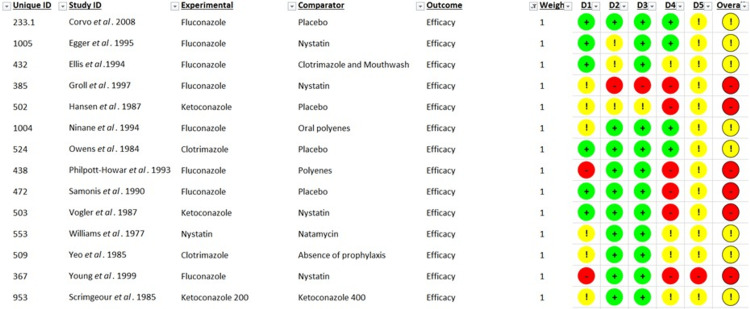
Risk of bias - prophylaxis

**Figure 4:**
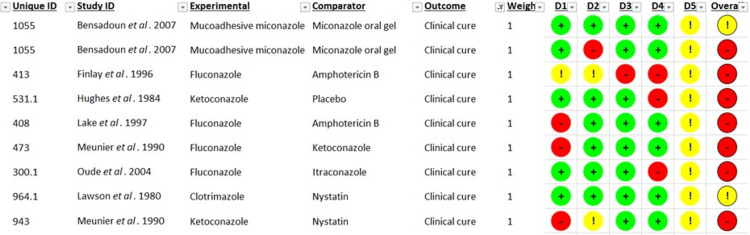
Risk of bias - clinical cure

**Figure 5:**
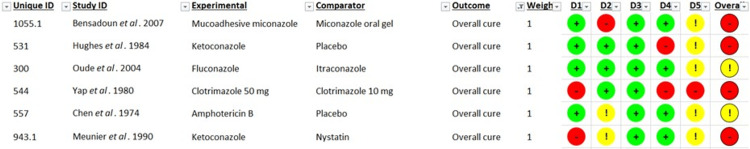
Risk of bias - overall cure

**Figure 6:**
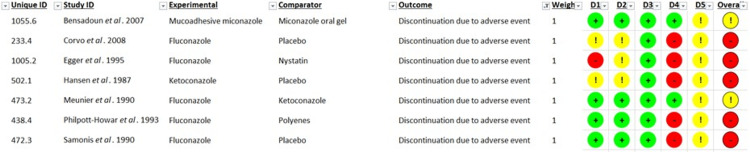
Risk of bias - discontinuation due to adverse events

**Figure 7:**
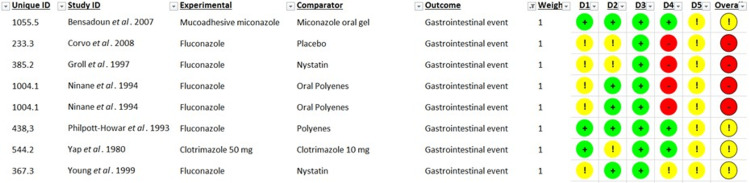
Risk of bias - gastrointestinal events (safety)

**Figure 8:**
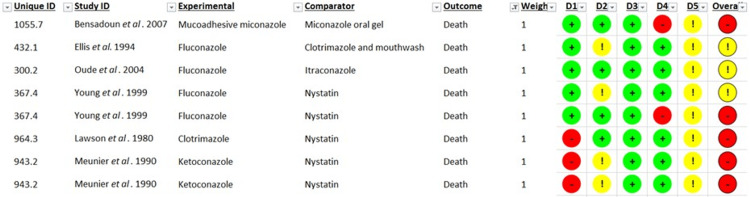
Risk of bias - death

## Appendices

Search strategy

**Table 6:** Search strategy for each database

Search	Query
PubMed	"Candidiasis, Oral"[MeSH Terms] OR Thrush[Title/Abstract] OR "Oral Moniliasis"[Title/Abstract] OR "Oral Moniliasis"[Title/Abstract] OR ((oropharyngeal[TIAB] OR oesophageal[TIAB] OR esophageal[TIAB] OR oropharynx[TIAB] OR Oropharynx[MH]) AND (fungal[TIAB] OR fungus[TIAB] OR candid*[TIAB])) OR (oral[TIAB] AND candid*[TIAB]) AND “Antifungal Agents”[MH] OR “Therapeutic Fungicides”[TIAB] OR Antifungal*[TIAB] OR Triazoles[MH] OR Triazole*[TIAB] OR Azoles[MH] OR Azole*[TIAB] OR Polyenes[MH] OR Polyene*[TIAB] OR Echinocandins[MH] OR Echinocandin*[TIAB] OR Mulundocandins[TIAB] OR Aculeacin*[TIAB] OR Pneumocandin*[TIAB] OR Allylamine[MH] OR Allylamine[TIAB] OR 3-Aminopropylene[TIAB] OR “3 Aminopropylene”[TIAB] OR Nystatin[MH] OR Nystatin[TIAB] OR Fungicidin[TIAB] OR Mycostatin[TIAB] OR Stamicin[TIAB] OR Stamycin[TIAB] OR Nilstat[TIAB] OR “Amphotericin B”[MH] OR Amphotericin[TIAB] OR Fungizone[TIAB] OR Amphocil[TIAB] OR Miconazole[MH] OR Miconazole[TIAB] OR “Miconasil Nitrate”[TIAB] OR Monistat[TIAB] OR Brentan[TIAB] OR Dactarin[TIAB] OR Clotrimazole[MH] OR Clotrimazole[TIAB] OR Klotrimazole[TIAB] OR Mycelex[TIAB] OR Lotrimin[TIAB] OR Canesten[TIAB] OR Kanesten[TIAB] OR Ketoconazole[MH] OR Ketoconazole[TIAB] OR Nizoral[TIAB] OR Itraconazole[MH] OR Itraconazole[TIAB] OR Sporanox[TIAB] OR Orungal[TIAB] OR Fluconazole[MH] OR Fluconazole[TIAB] OR Zonal[TIAB] OR Béagyne[TIAB] OR Diflucan[TIAB] OR “Fluc Hexal”[TIAB] OR Flucobeta[TIAB] OR FlucoLich[TIAB] OR Flunazul[TIAB] OR Fungata[TIAB] OR Lavisa[TIAB] OR Loitin[TIAB] OR Neofomiral[TIAB] OR Oxifungol[TIAB] OR Solacap[TIAB] OR Triflucan[TIAB] OR Voriconazole[MH] OR Voriconazole[TIAB] OR Vfend[TIAB] OR isavuconazole[Supplementary Concept] OR isavuconazole[TIAB] OR Cresemba[TIAB] OR ravuconazole[TIAB] OR posaconazole[Supplementary Concept] OR Posaconazole[TIAB] OR Noxafil[TIAB] OR Anidulafungin[MH] OR Anidulafungin[TIAB] OR Eraxis[TIAB] OR Caspofungin[MH] OR Caspofungin[TIAB] OR Cancidas[TIAB] OR Micafungin[MH] OR Micafungin[TIAB] OR Mycamine[TIAB] AND ((clinical[Title/Abstract] AND trial[Title/Abstract]) OR clinical trials as topic[MeSH Terms] OR clinical trial[Publication Type] OR random*[Title/Abstract] OR random allocation[MeSH Terms] OR therapeutic use[MeSH Subheading]) AND Neoplasms[MH] OR Neoplasm*[TIAB] OR Tumor*[TIAB] OR Neoplasia*[TIAB] OR Cancer*[TIAB] OR Malignanc*[TIAB] OR Malignant[TIAB] OR Oncologic[TIAB]
Web of Science	TS=(Thrush OR "Oral Moniliases" OR "Oral Moniliasis" OR ((oropharyngeal OR oesophageal OR esophageal OR oropharynx) AND (fungal OR fungus OR candid*)) OR (oral AND candid*)) AND TS=(“Therapeutic Fungicides” OR Antifungal* OR Triazole* OR Azole* OR Polyene* OR Echinocandin* OR Mulundocandins OR Aculeacin* OR Pneumocandin* OR Allylamine OR 3-Aminopropylene OR “3 Aminopropylene” OR Nystatin OR Fungicidin OR Mycostatin OR Stamicin OR Stamycin OR Nilstat OR Amphotericin OR Fungizone OR Amphocil OR Miconazole OR “Miconasil Nitrate” OR Monistat OR Brentan OR Dactarin OR Clotrimazole OR Klotrimazole OR Mycelex OR Lotrimin OR Canesten OR Kanesten OR Ketoconazole OR Nizoral OR Itraconazole OR Sporanox OR Orungal OR Fluconazole OR Zonal OR Béagyne OR Diflucan OR “Fluc Hexal” OR Flucobeta OR FlucoLich OR Flunazul OR Fungata OR Lavisa OR Loitin OR Neofomiral OR Oxifungol OR Solacap OR Triflucan OR Voriconazole OR Vfend OR isavuconazole OR Cresemba OR ravuconazole OR Posaconazole OR Noxafil OR Anidulafungin OR Eraxis OR Caspofungin OR Cancidas OR Micafungin OR Mycamine) AND TITLE-ABS-KEY((clinical AND trial) OR random*) AND TITLE-ABS-KEY(Neoplasm* OR Tumor* OR Neoplasia* OR Cancer* OR Malignanc* OR Malignant OR Oncologic)
Scopus	TITLE-ABS-KEY(Thrush OR "Oral Moniliasis" OR "Oral Moniliasis" OR ((oropharyngeal OR oesophageal OR esophageal OR oropharynx) AND (fungal OR fungus OR candid*)) OR (oral AND candid*)) AND TITLE-ABS-KEY(“Therapeutic Fungicides” OR Antifungal* OR Triazole* OR Azole* OR Polyene* OR Echinocandin* OR Mulundocandins OR Aculeacin* OR Pneumocandin* OR Allylamine OR 3-Aminopropylene OR “3 Aminopropylene” OR Nystatin OR Fungicidin OR Mycostatin OR Stamicin OR Stamycin OR Nilstat OR Amphotericin OR Fungizone OR Amphocil OR Miconazole OR “Miconasil Nitrate” OR Monistat OR Brentan OR Dactarin OR Clotrimazole OR Klotrimazole OR Mycelex OR Lotrimin OR Canesten OR Kanesten OR Ketoconazole OR Nizoral OR Itraconazole OR Sporanox OR Orungal OR Fluconazole OR Zonal OR Béagyne OR Diflucan OR “Fluc Hexal” OR Flucobeta OR FlucoLich OR Flunazul OR Fungata OR Lavisa OR Loitin OR Neofomiral OR Oxifungol OR Solacap OR Triflucan OR Voriconazole OR Vfend OR isavuconazole OR Cresemba OR ravuconazole OR Posaconazole OR Noxafil OR Anidulafungin OR Eraxis OR Caspofungin OR Cancidas OR Micafungin OR Mycamine)

List of included and excluded articles

**Table 7:** List of included articles

Author	Year	Title	Criteria
Bensadoun et al.	2007	Comparison of the efficacy and safety of miconazole 50 mg mucoadhesive buccal tablets to those of miconazole 500 mg gel in the treatment of oropharyngeal candidiasis: a prospective, randomised, single blind, multicenter, comparative, phase III trial in patients treated with radiotherapy for head and neck cancer	Included
Chen et al.	1974	Oral monilia study on patients with head and neck cancer during radiotherapy	Included
Corvò et al.	2008	Effects of fluconazole in the prophylaxis of oropharyngeal candidiasis in patients undergoing radiotherapy for head and neck tumour: results from a double-blind placebo-controlled trial	Included
Egger et al.	1995	Comparison of Fluconazole with Oral Polyenes in the Prevention of Fungal-Infections in Neutropenic Patients - a Prospective, Randomized, Single-Center Study	Included
Ellis et al.	1994	Controlled study of fluconazole in the prevention of fungal infections in neutropenic patients with haematological malignancies and bone marrow transplant recipients	Included
Finlay et al.	1996	A comparative study of the efficacy of fluconazole and amphotericin B in the treatment of oropharyngeal candidosis in patients undergoing radiotherapy for head and neck tumours	Included
Groll et al.	1997	Fluconazole versus nystatin in the prevention of candida infections in children and adolescents undergoing remission induction or consolidation chemotherapy for cancer	Included
Hansen et al.	1987	Ketoconazole in the prevention of candidiasis in patients with cancer. A prospective, randomized, controlled, double-blind study	Included
Hughes et al.	1983	Ketoconazole and candidiasis: a controlled study	Included
Lake et al.	1996	Fluconazole versus amphotericin B in the treatment of esophageal candidiasis in cancer patients	Included
Lawson et al.	1980	Comparison of clotrimazole troche and nystatin vaginal tablet in the treatment of oropharyngeal candidiasis	Included
Meunier, F et al.	1990	Therapy for oropharyngeal candidiasis in the immunocompromised host: a randomized double-blind study of fluconazole vs. ketoconazole	Included
Ninane et al.	1994	A Multicenter Study of Fluconazole Versus Oral Polyenes in the Prevention of Fungal Infection in Children with Hematological or Oncological Malignancies	Included
Oude et al.	2004	An open multicentre comparative study of the efficacy, safety and tolerance of fluconazole and itraconazole in the treatment of cancer patients with oropharyngeal candidiasis	Included
Owens et al.	1984	Prophylaxis of oral candidiasis with clotrimazole troches	Included
Philpott-Howard et al.	1993	Randomized comparison of oral fluconazole versus oral polyenes for the prevention of fungal infection in patients at risk of neutropenia. Multicentre Study Group	Included
Samonis et al.	1990	Prophylaxis of oropharyngeal candidiasis with fluconazole	Included
Scrimgeour et al.	1985	Ketoconazole prophylaxis in patients with solid tumours receiving aggressive immunosuppressive therapy. An open randomized comparison between 200 mg/d and 400 mg/d doses	Included
Vogler et al.	1987	A randomized trial comparing ketoconazole and nystatin prophylactic therapy in neutropenic patients	Included
Williams et al.	1977	Oral anticandidal prophylaxis in patients undergoing chemotherapy for acut- leukemia	Included
Yap et al.	1979	Oropharyngeal candidiasis treated with a troche form of clotrimazole	Included
Yeo et al.	1985	Prophylaxis of oropharyngeal candidiasis with clotrimazole	Included
Young et al.	1999	A double-blind comparison of fluconazole and nystatin in the prevention of candidiasis in patients with leukaemia. Antifungal Prophylaxis Study Group	Included
Meunier et al.	1990	Oral Treatment of Oropharyngeal Candidiasis with Nystatin Versus Ketoconazole in Cancer Patients: A Randomised Study	Included

**Table 8:** List of excluded studies with reasons for exclusion

Author	Year	Title	Criteria
Ardizzoni et al.	1996	Phase I study of simultaneous dose escalation and schedule acceleration of cyclophosphamide-doxorubicin-etoposide using granulocyte colony-stimulating factor with or without antimicrobial prophylaxis in patients with small-cell lung cancer	Population
Bagg, J. et al.	2003	High prevalence of non-albicans yeasts and detection of anti-fungal resistance in the oral flora of patients with advanced cancer	Population
Bagg, J.; Sweeney, M. P.	2003	Oral problems in advanced cancer	Population
Bensadoun, R. et al.	2006	A controlled, multicenter randomised phase 3 study of an extended release miconazole (50mg) bioadhesive buccal tablet (miconazole Lauriad (R)) once daily for local treatment of oropharyngeal candidiasis in head and neck cancer patients undergoing radiation therapy	Population
Bensadoun, R. et al.	2008	Comparison of the efficacy and safety of miconazole 50-mg mucoadhesive buccal tablets with miconazole 500-mg gel in the treatment of oropharyngeal candidiasis: a prospective, randomized, single-blind, multicenter, comparative, phase III trial in patients treated with radiotherapy for head and neck cancer	Population
Bodey et al.	1994	Antifungal prophylaxis during remission induction therapy for acute leukemia fluconazole versus intravenous amphotericin B	Population
Bodey, G. et al.	1982	Effect of systemic antimicrobial prophylaxis on microbial flora	Population
Boogaerts et al.	2001	Intravenous and oral itraconazole versus intravenous amphotericin B deoxycholate as empirical antifungal therapy for persistent fever in neutropenic patients with cancer who are receiving broad-spectrum antibacterial therapy - A randomized, controlled trial	Population
Braun, W. et al.	1972	[Candidiasis simulating chronic lichenoid pityriasis with perleche, following soor granuloma and generalized candidiasis]	Population
Brincker, H.	1977	Miconazole in oral candidiasis	Population
Brincker, H.	1978	Prophylactic treatment with miconazole in patients highly predisposed to fungal infection. A placebo-controlled double-blind study	Population
Conrad et al.	1990	Comparitive evaluation on nystatin pastille and clotrimazole troche for the treatment of candidal stomatitis in immunocompromised patients	Population
D'Antonio, D. et al.	1996	Effect of the current antimicrobial therapeutic strategy on fungal colonization in patients with hematologic malignancies	Population
Andrade, R. C. D. V. et al.	2022	Comparative randomized trial study about the efficacy of photobiomodulation and curcumin antimicrobial photodynamic therapy as a coadjuvant treatment of oral mucositis in oncologic patients: antimicrobial, analgesic, and degree alteration effect	Population
De Rosa, F. G. et al.	2021	Invasive Candidiasis in Patients with Solid Tumors Treated with Anidulafungin: A Post Hoc Analysis of Efficacy and Safety of Six Pooled Studies	Population
Vries-Hospers, H. G. et al.	1982	The effect of amphotericin B lozenges on the presence and number of Candida cells in the oropharynx of neutropenic leukemia patients	Population
DeGregorio, M. W. et al.	1982	Candida infections in patients with acute leukemia: ineffectiveness of nystatin prophylaxis and relationship between oropharyngeal and systemic candidiasis	Population
Dokos, C. et al.	2012	Pharmacokinetics, safety and efficacy of voriconazole in pediatric patients: An update	Population
Donnelly, J. P. et al.	1984	Oral ketoconazole and amphotericin B for the prevention of yeast colonization in patients with acute leukaemia	Population
Döring, M. et al.	2017	Efficacy, safety and feasibility of antifungal prophylaxis with posaconazole tablet in paediatric patients after haematopoietic stem cell transplantation	Population
Epstein, J. B. et al.	2003	Oral candidiasis in hematopoietic cell transplantation patients: an outcome-based analysis	Population
Fainstein, V. et al.	1987	Amphotericin B or ketoconazole therapy of fungal infections in neutropenic cancer patients	Population
Flynn, P. M. et al.	1995	Oropharyngeal candidiasis in immunocompromised children: a randomized, multicenter study of orally administered fluconazole suspension versus nystatin. The Multicenter Fluconazole Study Group	Population
Grigull, L. et al.	2007	Intravenous and oral sequential itraconazole antifungal prophylaxis in paediatric stem cell transplantation recipients: A pilot study for evaluation of safety and efficacy	Population
Günther, I. et al.	1986	Fungal infections in acute leukemia patients during selective decontamination of the digestive tract--a clinical, laboratory and pathological study	Population
Harousseau, J. L. et al.	2000	Itraconazole oral solution for primary prophylaxis of fungal infections in patients with hematological malignancy and profound neutropenia: A randomized, double-blind, double-placebo, multicenter trial comparing itraconazole and amphotericin B	Population
Hoppe, J. E. et al.	1995	Orointestinal yeast colonization of paediatric oncologic patients during antifungal prophylaxis: results of quantitative culture and Candida serology and comparison of three polyenes	Population
Huijgens, P. C. et al.	1999	Fluconazole versus itraconazole for the prevention of fungal infections in haemato-oncology	Population
Huijgens, P. C. et al.	1993	The prophylactic use of fluconazole 50 vs. 100 mg daily in haematological malignancies	Population
Iriyama, N. et al.	2011	Efficacy and safety of antifungal prophylaxis with oral itraconazole solution among patients receiving corticosteroids: who should be given prophylaxis?	Population
Katragkou, A. et al.	2012	Posaconazole: When and how? The clinician's view	Population
Koc, M.; Aktas, E.	2003	Prophylactic treatment of mycotic mucositis in radiotherapy of patients with head and neck cancers	Population
Laverdière, M. et al.	2000	Impact of fluconazole prophylaxis on fungal colonization and infection rates in neutropenic patients. The Canadian Fluconazole Study	Population
Lefebvre, J. L. et al.	2002	A comparative study of the efficacy and safety of fluconazole oral suspension and amphotericin B oral suspension in cancer patients with mucositis	Population
Matthews, R. H.; Ercal, N.	1996	Prevention of mucositis in irradiated head and neck cancer patients	Population
Menichetti, F. et al.	1999	Itraconazole oral solution as prophylaxis for fungal infections in neutropenic patients with hematologic malignancies: a randomized, placebo-controlled, double-blind, multicenter trial. GIMEMA Infection Program. Gruppo Italiano Malattie Ematologiche dell' Adulto	Population
Mirsky, H. S.; Cuttner, J.	1972	Fungal infection in acute leukemia	Population
Morgenstern, G. R. et al.	1999	A randomized controlled trial of itraconazole versus fluconazole for the prevention of fungal infections in patients with haematological malignancies. U.K. Multicentre Antifungal Prophylaxis Study Group	Population
Nicolatou-Galitis, O. et al.	2006	Effect of fluconazole antifungal prophylaxis on oral mucositis in head and neck cancer patients receiving radiotherapy	Population
Nucci, M. et al.	2000	A double-blind, randomized, placebo-controlled trial of itraconazole capsules as antifungal prophylaxis for neutropenic patients	Population
Pitten, F. A. et al.	2003	Do cancer patients with chemotherapy-induced leukopenia benefit from an antiseptic chlorhexidine-based oral rinse? A double-blind, block-randomized, controlled study	Population
Prentice, A. G.; Bradford, G. R.	1989	Prophylaxis of fungal infections with itraconazole during remission-induction therapy	Population
Quintiliani, R. et al.	1984	Treatment and prevention of oropharyngeal candidiasis	Population
Rao, S. P. et al.	1985	Candida esophagitis in two children with acute leukemia: successful therapy with ketoconazole	Population
Rozenberg-Arska, M. et al.	1991	A randomized study to compare oral fluconazole to amphotericin B in the prevention of fungal infections in patients with acute leukaemia	Population
Schaffner, A.; Schaffner, M.	1995	Effect of prophylactic fluconazole on the frequency of fungal infections, amphotericin B use, and health care costs in patients undergoing intensive chemotherapy for hematologic neoplasias	Population
Selvy, N. et al.	2010	[Prophylaxis use of posaconazole (P) in an hemato-oncology unit: a retrospective study]	Population
Vazquez, J. A. et al.	2008	A phase 2, open-label study of the safety and efficacy of intravenous anidulafungin as a treatment for azole-refractory mucosal candidiasis	Population
Vreugdenhil, G. et al.	1993	Efficacy of itraconazole in the prevention of fungal infections among neutropenic patients with hematologic malignancies and intensive chemotherapy. A double blind, placebo controlled study	Population
Winston, D. J. et al.	2011	Efficacy, safety, and breakthrough infections associated with standard long-term posaconazole antifungal prophylaxis in allogeneic stem cell transplantation recipients	Population
Winston, D. J. et al.	1993	Fluconazole prophylaxis of fungal infections in patients with acute leukemia: Results of a randomized placebo-controlled, double-blind, multicenter trial	Population
Yan, Z. et al.	2016	The Efficacy and Safety of Miconazole Nitrate Mucoadhesive Tablets versus Itraconazole Capsules in the Treatment of Oral Candidiasis: An Open-Label, Randomized, Multicenter Trial	Population
	2008	Miconazole mucoadhesive gingival tablet: Orpharyngeal candidiasis: New form of tablet with no proven advantages	Study desing
Aanpreung, P.; Veerakul, G.	1997	Itraconazole for treatment of oral candidosis in pediatric cancer patients	Study desing
Ahmadi, A.	2012	Potential prevention: Aloe vera mouthwash may reduce radiation-induced oral mucositis in head and neck cancer patients	Study desing
Akova, M. et al.	1994	Efficacy of fluconazole in the treatment of upper gastrointestinal candidiasis in neutropenic patients with cancer: factors influencing the outcome	Study desing
Akpan, A.; Morgan, R.	2002	Oral candidiasis	Study desing
Al-Abeid, H. M. et al.	2004	Isolation and characterization of Candida spp. in Jordanian cancer patients: prevalence, pathogenic determinants, and antifungal sensitivity	Study desing
Avilés, A.	1987	Clotrimazole treatment for prevention of oral candidiasis in patients with acute leukemia undergoing chemotherapy	Study desing
Barrett, A. P.	1984	Evaluation of nystatin in prevention and elimination of oropharyngeal Candida in immunosuppressed patients	Study desing
Belazi, M. et al.	2004	Oral Candida isolates in patients undergoing radiotherapy for head and neck cancer: prevalence, azole susceptibility profiles and response to antifungal treatment	Study desing
Bensadoun, R. J.	2005	A controlled, multicenter randomised phase 3 study of an extended release miconazole (50 mg) bioadhesive buccal tablet (miconazole Lauriad (R)) once daily for local treatment of oropharyngeal candidiasis in head and neck cancer patients undergoing radiation therapy	Study desing
Bensadoun, R. J. et al.	2005	A controlled, multicenter randomised phase 3 study of an extended release miconazole (50mg) bioadhesive buccal tablet once daily for local treatment of oropharyngeal candidiasis in head and neck cancer patients undergoing radiation therapy	Study desing
Berkowitz, R. J. et al.	1985	Oropharyngeal Candida prophylaxis in pediatric bone marrow transplant patients	Study desing
Brincker, H.	1976	Treatment of oral candidiasis in debilitated patients with miconazole--a new potent antifungal drug	Study desing
Candoni, A. et al.	2007	Intravenous itraconazole: Use in allogenic bone marrow transplantation and acute leukaemias	Study desing
Čáp, J. et al.	1993	Fluconazole in children: First experience with prophylaxis in chemotherapy- induced neutropenia in pediatric patients with cancer	Study desing
Chandrasekar, P. H.; Gatny, C. M.	1994	The effect of fluconazole prophylaxis on fungal colonization in neutropenic cancer patients. Bone Marrow Transplantation Team	Study desing
Collins, C. D. et al.	2011	Management of oropharyngeal candidiasis with localized oral miconazole therapy: Efficacy, safety, and patient acceptability	Study desing
Cornely, O. A. et al.	2001	[Antifungal prophylaxis in neutropenic patients]	Study desing
Crowe, H. M.	1990	Focus on fluconazole: A potent antifungal agent	Study desing
Cuttner, J. et al.	1986	Clotrimazole treatment for prevention of oral candidiasis in patients with acute leukemia undergoing chemotherapy. Results of a double-blind study	Study desing
Dunsche, A. et al.	1994	[Indication for and effectiveness of fluconazole in oral candidiasis]	Study desing
Ehninger, G. et al.	1996	Fluconazole in the prophylaxis of fungal infection after bone marrow transplantation	Study desing
Epstein, D. J. et al.	2018	Echinocandin prophylaxis in patients undergoing haematopoietic cell transplantation and other treatments for haematological malignancies	Study desing
Epstein, J. B.	1989	Oral and pharyngeal candidiasis. Topical agents for management and prevention	Study desing
Epstein, J. B. et al.	1996	Prophylaxis of candidiasis in patients with leukemia and bone marrow transplants	Study desing
Evans, W. K. et al.	1987	Nystatin lozenges: A useful treatment for oral candidiasis in cancer patients	Study desing
Eyre, J.; Nally, F. F.	1971	Oral candidosis and carcinoma	Study desing
Fasano, C. et al.	1994	Fluconazole treatment of children with severe fungal infections not treatable with conventional agents	Study desing
Gonzalez, G.	1971	Esophageal moniliasis	Study desing
Goranov, S. et al.	1999	Antifungal prophylaxis with low doses fluconazole in patients with hematological malignancies	Study desing
Groll, A. et al.	1992	Fluconazole treatment of oropharyngeal candidosis in spediatric cancer patients with severe mucositis following antineoplastic chemotherapy	Study desing
Holst, E.	1984	Natamycin and nystatin for treatment of oral candidiasis during and after radiotherapy	Study desing
Jenkins, W. M. et al.	1973	Oral infections with Candida albicans	Study desing
Kauffman, C. A. et al.	1984	Effect of prophylactic ketoconazole and nystatin on fungal flora	Study desing
Krcméry, V., Jr. et al.	1991	Fluconazole in the treatment of mycotic oropharyngeal stomatitis and esophagitis in neutropenic cancer patients	Study desing
Lagman, R. et al.	2017	Single-Dose Fluconazole Therapy for Oral Thrush in Hospice and Palliative Medicine Patients	Study desing
Lass-Flörl, C. et al.	2003	Fungal colonization in neutropenic patients: a randomized study comparing itraconazole solution and amphotericin B solution	Study desing
Malcom, L. G. et al.	1982	A phase III trial comparing ketoconazole (R41400) and nystatin in the prevention of oral candidiasis and invasive fungal infections in the neutropenic immunosuppressed patient	Study desing
Mensa, J. et al.	2009	Treatment of fungal infections in patients with hematologic neoplasia	Study desing
Meunier, F.	1990	Fluconazole treatment of fungal infections in the immunocompromised host	Study desing
Meunier, F. et al.	1994	Value of antifungal prophylaxis with antifungal drugs against oropharyngeal candidiasis in cancer patients	Study desing
Myoken, Y. et al.	2004	Oropharyngeal Candida colonization and infection in neutropenic patients with hematologic malignancies	Study desing
Olsen, I.; Pedersen, K. N.	1972	[Oral candidosis]	Study desing
Palmblad, J. et al.	1992	Oral ketoconazole prophylaxis for Candida infections during induction therapy for acute leukaemia in adults: more bacteraemias	Study desing
Redding, S. W. et al.	2004	Candida glabrata is an emerging cause of oropharyngeal candidiasis in patients receiving radiation for head and neck cancer	Study desing
Redding, S. W. et al.	2002	Candida glabrata oropharyngeal candidiasis in patients receiving radiation treatment for head and neck cancer	Study desing
Shechtman, L. B. et al.	1984	Clotrimazole treatment of oral candidiasis in patients with neoplastic disease	Study desing
Soutome, S. et al.	2022	A preliminary study of suppression of candida infection by miconazole mucoadhesive tablets in oral or oropharyngeal cancer patients undergoing radiotherapy	Study desing
Stark, A. et al.	1993	Fluconazole chemoprophylaxis in neutropenic patients	Study desing
Stuchlik, S.	1975	[Treatment of mouth candidiasis in leukemia patients with parenterally administered miconazole]	Study desing
Studena, V. et al.	1995	Fluconazole versus itraconazole in therapy of oropharyngeal candidiasis in cancer patients: a prospective comparative randomized trial	Study desing
Tam, J. Y. et al.	1992	Prophylactic fluconazole and candida krusei infections	Study desing
Viscoli, C. et al.	1991	Fluconazole in the treatment of candidiasis in immunocompromised children	Study desing
Viscoli, C.; Castagnola, E.	1998	Planned progressive antimicrobial therapy in neutropenic patients	Study desing
Williams, C. J.	1978	Nystatin prophylaxis in leukemia and lymphoma	Study desing
Worthington, H. V.; Clarkson, J. E.	2002	Prevention of oral mucositis and oral candidiasis for patients with cancer treated with chemotherapy: cochrane systematic review	Study desing
Worthington, H. V. et al.	2002	Interventions for preventing oral candidiasis for patients with cancer receiving treatment	Study desing
Worthington, H. V. et al.	2007	Interventions for treating oral candidiasis for patients with cancer receiving treatment	Study desing
Worthington, H. V. et al.	2010	Interventions for treating oral candidiasis for patients with cancer receiving treatment	Study desing
Worthington, H. V. et al.	2004	Interventions for preventing oral candidiasis for patients with cancer receiving treatment	Study desing
Barantsevich et al.	1992	[Esophageal candidiasis in patients with hemoblastosis]	Language
Mitrokhin et al.	2003	[Itraconazole (Orungal) in the treatment of mycotic infections in oncologic patients]	Language
Ohno, T. et al.	2012	[Effectiveness of antifungal agents for oral candidiasis in inpatients]	Language
Toubai, T. et al.	2003	Effect of prophylaxis against myocosis in patients with hematological malignancy disease: Efficacy of dosage of itraconazole	Language
Tsuyuki, S. et al.	2012	[Usefulness of antimycotic agents (itraconazole) in chemotherapy-induced mucositis of breast cancer patients]	Language
Wang, J. Z. et al.	2005	Efficacy of intravenous administration of itraconazole on fungal infections in immunocompromised patients with hematological diseases	Language

Risk of bias of the included studies (per outcome)

The RoB 2.0 tool was applied according to Cochrane Collaboration recommendations and following the above mentioned detailed criteria. The overall risk of bias judgment for each study was determined based on the highest risk level identified in any domain. 

Selection Bias Domain

This assesses whether participants were randomly assigned to intervention or control groups, and if the allocation concealment process was adequate. For each study, we reviewed the method of random sequence generation and allocation concealment. Studies were rated as: Low risk: when randomization was adequately described, and allocation was concealed; Some concerns: if randomization was mentioned but not well described or if there was potential for allocation bias; High risk: when randomization was absent or clearly described as inadequate.

Performance Bias Domain

This evaluates if participants and personnel were blinded to the intervention. We, thus, checked the blinding methods described in each study. Studies were rated as: Low risk: if blinding of participants and personnel was clearly implemented; Some concerns: if there was partial blinding or blinding was not described clearly; High risk: if blinding was not performed or described as inadequate.

Detection Bias Domain

This assesses the blinding of outcome assessment. We reviewed how outcome assessors were blinded to the intervention. Studies were rated as: Low risk: when outcome assessors were blinded; Some concerns: if blinding was partial or not clearly described; High risk: if there was no blinding of outcome assessment or if it was described as inadequate.

Attrition Bias Domain

This examines how missing data and dropouts were handled. We, thus, assessed whether the study accounted for all participants and the reasons for dropouts. Studies were rated as: Low risk: if attrition was minimal and well-handled with appropriate analyses; Some concerns: if there was some loss to follow-up but with unclear impact on results; High risk: if there was significant attrition with potential impact on results or inadequate handling of missing data.

Reporting Bias Domain

This evaluates whether the study selectively reported outcomes. We checked for pre-specified outcomes and whether all were reported. Studies were rated as: Low risk: if outcomes were reported as planned with no selective reporting; Some concerns: if there was some discrepancy between reported and pre-specified outcomes; High risk: if selective reporting of outcomes was evident.

Ranking of treatment comparisons from the network meta-analysis

**Table 9:** Treatment ranking of the network meta-analysis Note: Data are presented as p-scores (%). A higher p-score indicates a greater probability that the treatment is associated with the outcome. AMB: Amphotericin B; AUS: Absence of treatment; CETO: Ketoconazole; FLU: Fluconazole; CLO: Clotrimazole; NATA: Natamicin; NIS: Nystatin; ITRA: Itraconazole; PLA: Placebo

Drug	Prophylaxis	Clinical cure	Overall cure
AMB	--	16%	79%
AUS	2%	--	--
CETO	65%	57%	53%
CLO	52%	77%	--
FLU	85%	67%	--
ITRA	--	41%	--
NATA	70%	--	--
NIS	54%	86%	64%
PLA	22%	5%	4%
